# HIV and Hepatitis C Mortality in Massachusetts, 2002–2011: Spatial Cluster and Trend Analysis of HIV and HCV Using Multiple Cause of Death

**DOI:** 10.1371/journal.pone.0114822

**Published:** 2014-12-11

**Authors:** David J. Meyers, Maria Elena Hood, Thomas J. Stopka

**Affiliations:** 1 Department of Health Policy and Management, Harvard School of Public Health, Boston, Massachusetts, United States of America; 2 Department of Public Health and Community Medicine, Tufts University School of Medicine, Boston, Massachusetts, United States of America; 3 Office of Data Management and Outcomes Assessment, Massachusetts Department of Public Health, Boston, Massachusetts, United States of America; Harvard Medical School, United States of America

## Abstract

**Background:**

Infectious diseases, while associated with a much smaller proportion of deaths than they were 50 years ago, still play a significant role in mortality across the state of Massachusetts. Most analysis of infectious disease mortality in the state only take into account the underlying cause of death, rather than contributing causes of death, which may not capture the full extent of mortality trends for infectious diseases such as HIV and the Hepatitis C virus (HCV).

**Methods:**

In this study we sought to evaluate current trends in infectious disease mortality across the state using a multiple cause of death methodology. We performed a mortality trend analysis, identified spatial clusters of disease using a 5-step geoprocessing approach and examined spatial-temporal clustering trends in infectious disease mortality in Massachusetts from 2002–2011, with a focus on HIV/AIDS and HCV.

**Results:**

Significant clusters of high infectious disease mortality in space and time throughout the state were detected through both spatial and space time cluster analysis. The most significant clusters occurred in Springfield, Worcester, South Boston, the Merrimack Valley, and New Bedford with other smaller clusters detected across the state. Multiple cause of death mortality rates were much higher than underlying cause mortality alone, and significant disparities existed across race and age groups.

**Conclusions:**

We found that our multi-method analyses, which focused on contributing causes of death, were more robust than analyses that focused on underlying cause of death alone. Our results may be used to inform public health resource allocation for infectious disease prevention and treatment programs, provide novel insight into the current state of infectious disease mortality throughout the state, and benefited from approaches that may more accurately document mortality trends.

## Introduction

In 1842, infectious diseases were the leading causes of death in Massachusetts, accounting for 47% of all deaths while in 2011 infectious diseases accounted for about 3% of all deaths in the state [1]. However, there may be a recent increase in infectious diseases such as hepatitis, influenza/pneumonia and septicemia within subpopulations in some regions of the Commonwealth. Looking at underlying cause of death, in 2011, HIV/AIDS was the 29^th^ leading cause of death in the state [1]. Currently, mortality trends for these diseases are not routinely examined in Massachusetts, and they typically rely solely on the underlying cause of death, and there may be long term and cyclical patterns that are not being detected.

Human immunodeficiency virus-1 (HIV) is a virus that affects the immune system, which greatly increases the risk of those infected to contract opportunistic infections. While the HIV-1/AIDS epidemic hit its peak in the United States in the late 90s, and its incidence has been on the decline, its chronic nature and associated mortality rates still make it a major concern across the United States and Massachusetts [2].

Viral hepatitis is an infection of the liver caused by several different viruses in the United States [3]. Between 1999 and 2007, there has been an increase in recorded deaths caused by the hepatitis C virus (HCV) across the United States, and it still remains a rising health issue today [4]. Data on extent and spatial distribution of HCV mortality in the state of Massachusetts is limited, highlighting the utility of this research.

The goal of this study was to examine current geospatial and temporal trends in infectious disease mortality with a particular focus in HIV/AIDS and the HCV across Massachusetts using multiple methodologies.

## Methods

Since the primary source of data was for decedents, this study was determined to be "Not Human Subjects Research" by the Tufts University School of Medicine Institutional Review Board and the Privacy and Data Access Office of the Massachusetts Department of Public Health. All data were de-identified prior to access.

In order to examine current geospatial and temporal trends, we conducted three different epidemiologic analyses: a general mortality trend analysis, a spatial cluster (hotspot) analysis, and a spatial temporal cluster analysis. For this analysis, we focused on HCV and HIV/AIDS. Table 1 provides a list of each test we conducted with the corresponding data and statistical tool.

**Table 1:** Overview of analytical tools.

Research Question	Analysis	Data Sources	Analytical Program
Is HIV and HCV mortality significantly clustered in MA census tracts?	Getis Ord Gi* Hot-Spot Cluster Analysis	Massachusetts Deaths Database, Massachusetts Census Tract Shapefiles	ArcGIS 10.1, SAS 9.3, MS Excel 2013
How is HIV and HCV related mortality in Massachusetts distributed spatially?	Discrete Poisson Space-Time Model	Massachusetts Deaths Database, Massachusetts Census Tract Shapefiles	SatScan, SAS
Is HIV and HCV related mortality in Massachusetts clustered in space and time?	Discrete Poisson Spatial Variation in Temporal Trends Model	Massachusetts Deaths Database, Massachusetts Census Tract Shapefiles	SatScan, SAS

### Sources of Data and Software

#### Mortality Data

Data on mortality were based on information retrieved from death certificates filed with the Massachusetts Department of Public Health-Registry of Vital Records and Statistics. Physicians and medical examiners assign the cause of death through a system that allows for the possibility of multiple causes. Demographic information on the certificates, such as age, race, Hispanic ethnicity, gender, educational attainment, marital status, and occupation, is recorded by the funeral director based on information provided by an informant, usually a family member, or, in the absence of an informant, based on observation or omitted.

All data in this paper were for Massachusetts residents. Resident data include all deaths that occur to residents of the Commonwealth, regardless of where the deaths occur. In Massachusetts, a resident is a person with a permanent address in one of the 351 cities and towns. There is an exchange agreement among the 50 states, District of Columbia, Puerto Rico, US Virgin Islands, Guam, and Canadian provinces that provides for the exchange of copies of death records for persons dying in a state other than their state of residence.

Death certificates in Massachusetts allow for the selection of one underlying cause and up to 15 contributing causes of death. The underlying cause of death is generated by the Super Mortality Medical Indexing, Classification, and Retrieval system (Super MICAR). This is a computer software algorithm developed by the National Center for Health Statistics and used by all US jurisdictions so that the assignment of cause of death codes is consistent. While most analysis conducted on mortality trends are conducted using just the underlying cause, this may miss patterns that may exist when taking into consideration contributing cause of death [5]. Multiple cause of death analysis have been conducted with success in a variety of international settings, allowing for a better detection of underlying signal [6]–[8]. In order to identify these patterns in the data, we used multiple causes of death data for analysis.

For this study, we examined HIV-1 and HCV death data from 2002 to 2011. In order to accurately capture the mortality burden of these diseases in the state, we included all deaths with HIV-1 and HCV as either the underlying cause of death or any contributing cause of death (ICD-10: A00-B99, J17-J18) in the analysis. This dataset contained 4,148 records and 95 variables, which were then pared down to only deaths attributable to HCV and HIV/AIDS. The dataset included one underlying cause of death and up to 15 contributing causes of death for each decedent, as well as demographic information such as their age, race, gender, and nationality, among others. Additional variables were created to reclassify race into four mutually exclusive categories, and to recode the date of death into a format more easily read by the SatScan software. The dataset also contained the last six digits of the 11-digit census tract code for census tract of the decedent's residence which was used in spatial analysis. These data were divided and analyzed separately for each disease category of interest.

#### Geographic Data

We acquired geographic census tiger boundary data from MassGIS which provides updated versions of Massachusetts census tract shapefiles for public use [9]. These shapefiles contained an 11 digit census tract ID that was used to join the shapefiles with the aggregated mortality data, and also included 2010 US census population estimates for each census tract from the 2010 decennial census.

#### Population Data

At the state level, population estimates for each year by age, gender and race and ethnicity were provided by the Massachusetts Department of Public Health and were used when examining overall trends in mortality rates per 1,000 population for the state overall and for each of the major race groups. For the geographic components of our analysis, we were only able to use population estimates from the US census bureau's 2010 census. The 2010 data were generalized for the ten-year span in all geographic analyses.

#### Software

Primary data organization and analysis was conducted using SAS v9.3 (Cary, NC). Mapping and calculation of spatial clusters was conducted using Esri ArcGIS v10.1 (Redlands, CA). Spatial-Temporal clustering was assessed using SatScan v9.2 (Boston, MA). Additional data manipulation and visualization was conducted in Microsoft Excel.

### Statistical Analysis

#### Mortality Trend Analysis

In order to assess the general trends in mortality across each disease category, we calculated several sets of statistics. The number of deaths for each condition was summed by both underlying cause and contributing cause for each year in order to show change over time and the difference between using underlying cause as compared to a multiple cause methodology, which included contributing causes. Annual mortality rates were calculated using yearly population estimates. Age Adjusted Mortality Rates were then calculated by race with 95% confidence intervals, standardized to 2000 US population in order to assess whether any disparities existed. Age specific mortality rates were also calculated by age group and for each year by race. Due to concerns about systematic underreporting of HCV deaths on death certificates, we also estimated the number of HCV deaths annually based on unreported rates described by Mahajan et al. [10] who found in a large cohort study that only 19% of those with HCV had their HCV documented on their death certificate. To calculate our estimates, we assumed that our observed deaths represented just 19% of total deaths in the state and calculated what an estimated number of deaths might be if 100% of deaths were observed (Observed Count ×5.26).

#### Spatial Cluster Analysis

Hotspot cluster analysis is a useful method for determining where geographic clusters of disease exist [11]. These analyses have been conducted to find clusters of disease and mortality occurrence in a variety of settings [12]–[14]. Stopka et al. have described a five step process to detect granular clusters using a Getis-Ord Gi* test in an empirical manner [15]. This method was used to determine geographic patterns of HIV and HCV mortality in the current study.

Thematic maps were first created for each disease, aggregated to the census tract level, in order to examine descriptive geographic distributions. An animation was then created displaying each disease for each year in the ten year period in order to show any visual changes over time (data not shown).

To perform the hotspot cluster analysis following the 5-step geoprocessing approach used by Stopka et al., the first step was to control for variability in census tract areal size. To do this we removed census tracts, which had a square mile area greater than 1.5 standard deviations above the mean area, and island tracts, in order to focus on contiguous tracts of a homogeneous size. Next, to begin to define the appropriate spatial scale for cluster analyses, we calculated the average and maximum distance between any given census tract and its 2 closest neighbors. We used these distances to conduct incremental spatial autocorrelations, which runs 30 repetitions of a Moran's I test, in order to determine the spatial scale at which the data displayed the most intense clustering of disease. This spatial scale was then used to create a spatial weights matrix to weight each census tract based on its size and location within the dataset. Finally, we performed the Getis-Ord GI* hotspot cluster analyses in ArcGIS to identify statistically significant clusters of census tracts with higher (hotspots) and lower (coldspots) than expected HIV/AIDS and HCV mortality rates, when compared to all census tracts in Massachusetts.

#### Spatio-Temporal Cluster Analysis

We used SaTScan to assess geospatial and temporal clustering. SaTScan performs tests to detect clusters in both time and space through different probability models depending on the composition of the dataset [16]. Spatial and temporal clustering has been useful in determining patterns in mortality in other settings and is valuable as the added time dimension can provide additional insight into geographic trends of disease [17]–[19].

First, we used a discrete Poisson model to calculate space-time clusters, which are locations (i.e., circular polygons) that displayed higher than expected mortality for HIV/AIDS or HCV during a specific time frame. The returned data included the relative risk of mortality for the selected disease in that cluster compared to risk in other census tracts outside of the cluster. Next, we calculated trends in spatial-temporal clusters using a discrete Poisson model to determine if there was a significant change over time of mortality in a given cluster as compared to the rest of the state.

For all disease conditions, we selected a scanning window a priori that comprised “5% of the population at risk” within the study area. This scanning window places a limit on the maximum amount of the population that can be included in a cluster of disease. In order to produce the most granular results, we selected a smaller scanning window; however there is no defined method in selecting this scanning window. Additionally, we aggregated data in 3 month periods within SatScan to facilitate more efficient analysis. For HIV/AIDS and HCV, we used a three month time period of aggregation over the ten year time frame of the study to assess any long term patterns in the clustering of disease.

## Results

In total there were a total of 4,818 deaths associated with HIV/AIDS and HCV for the whole period of 2002–2011: 2,913 deaths associated with HCV and 1,905 deaths associated with HIV/AIDS.

### Mortality Trend Analysis

**Figure 1:**
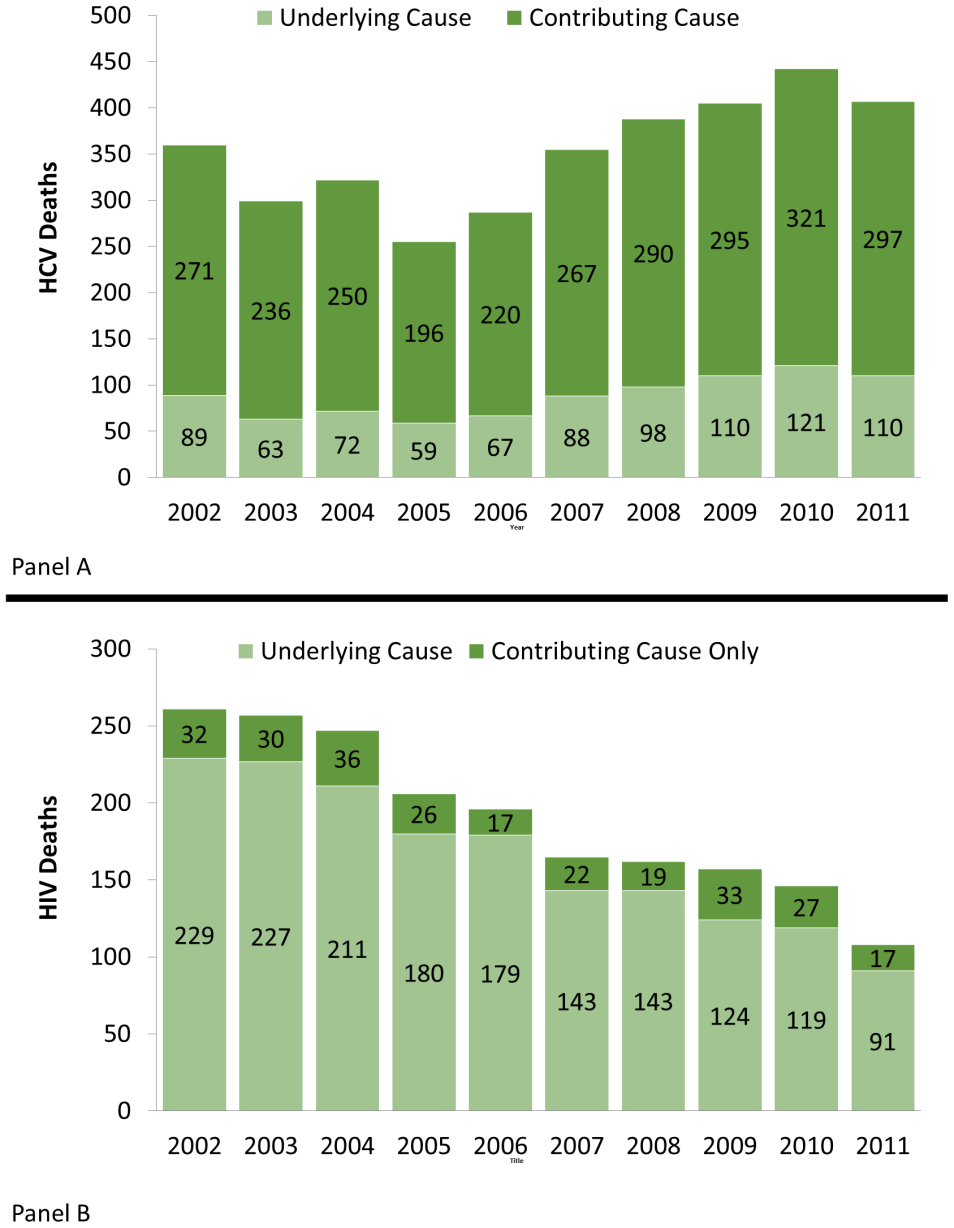
Hepatitis C and HIV/AIDS Death Totals by Year in MA between 2002–2011. Panel A is Hepatitis C. Panel B is HIV/AIDS.

**Figure 2:**
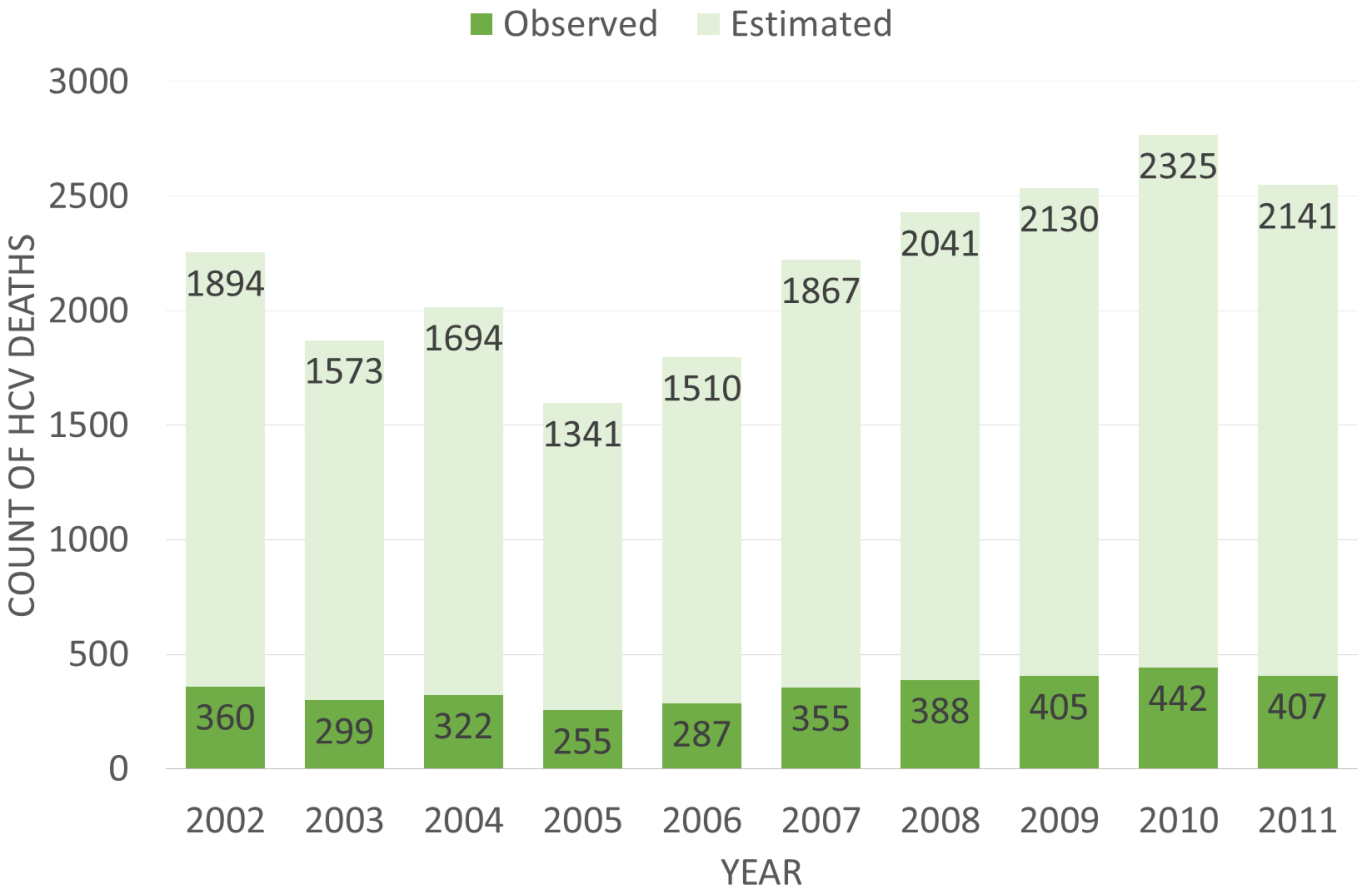
Observed and Estimated Total HCV Deaths per Year. The observed counts are the totals of both contributing and underlying cause attributed HCV deaths. The estimated is based off the assumption that only 19% of HCV cases are recorded from death certificates and is the estimated total if 100% were to be counted.

Fig. 1 Panel A and B show the total number of deaths each year for HCV and HIV/AIDS respectively with the number of Underlying Cause Deaths and the number of Contributing Cause Deaths delineated. Between 2002 and 2011, HCV mortality remained relatively constant and was largely classified as a contributing cause of death. HIV/AIDS mortality conversely declined and was primarily classified as an underlying cause of death. Fig. 2 shows the total observed deaths with HCV as a contributing or underlying cause and an estimated annual number of deaths based on the work of Mahajan et al. [10].

**Figure 3:**
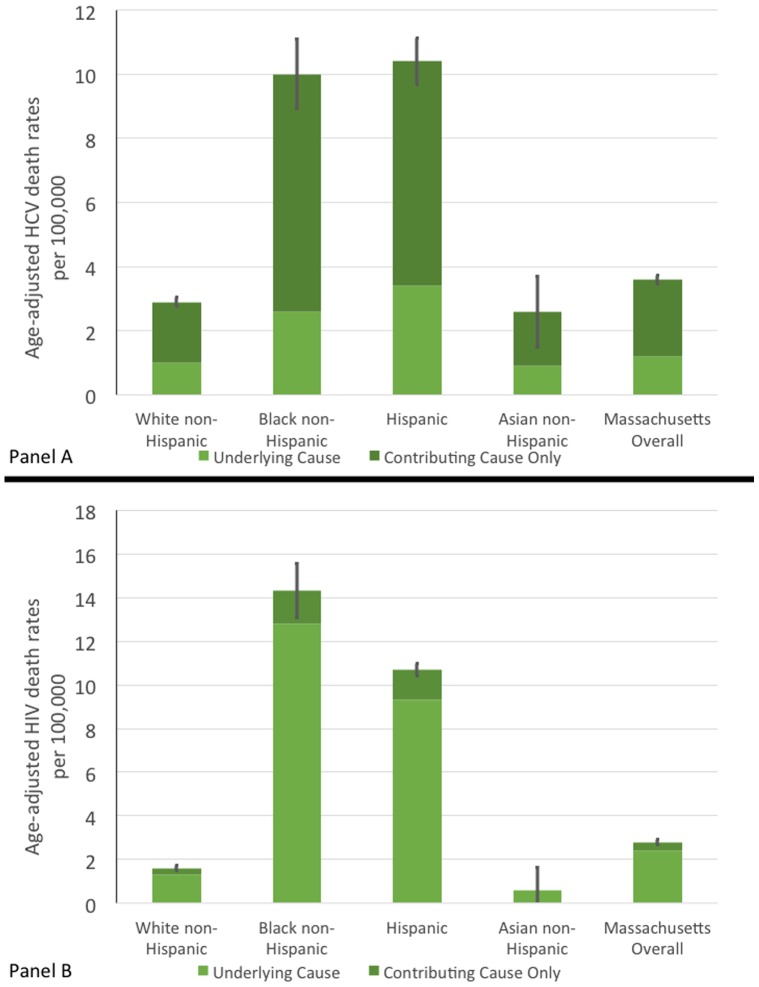
Standardized HCV and HIV Mortality Rates by Race in MA between 2002–2011. Panel A is Hepatitis C. Panel B is HIV/AIDS.

Fig. 3, Panels A and B display standardized age-adjusted mortality rates per 100,000 people for HCV and HIV/AIDS respectively by race and Hispanic ethnicity. Each graph also includes error bars that illustrate the 95% confidence interval of the overall mortality rate, which included contributing and underlying cause of death. For both diseases, there exists a similar disparity in the mortality rates between White and Asian populations and Black and Hispanic populations, which had significantly higher rates. Death rates among Blacks and Hispanics are significantly higher than those of White and Asian populations for HIV/AIDS and HCV.

**Figure 4:**
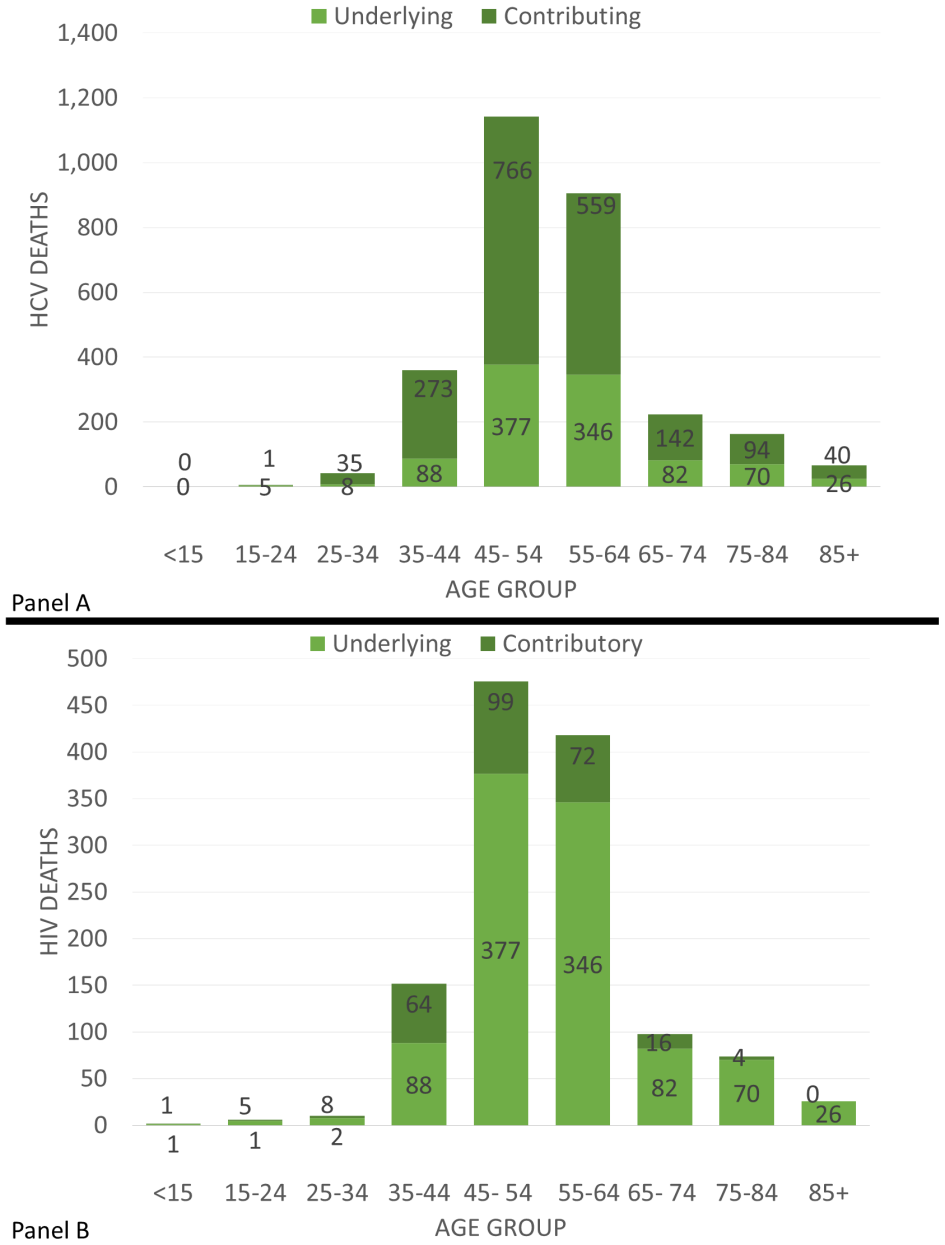
HCV and HIV/AIDS Deaths by Age in MA between 2002–2011. Panel A is Hepatitis C. Panel B is HIV/AIDS.

Fig. 4, Panels A and B display the age distribution of deaths for HCV and HIV/AIDS respectively over the ten-year period. In both, deaths appear to peak between 45–64 years of age.

**Figure 5:**
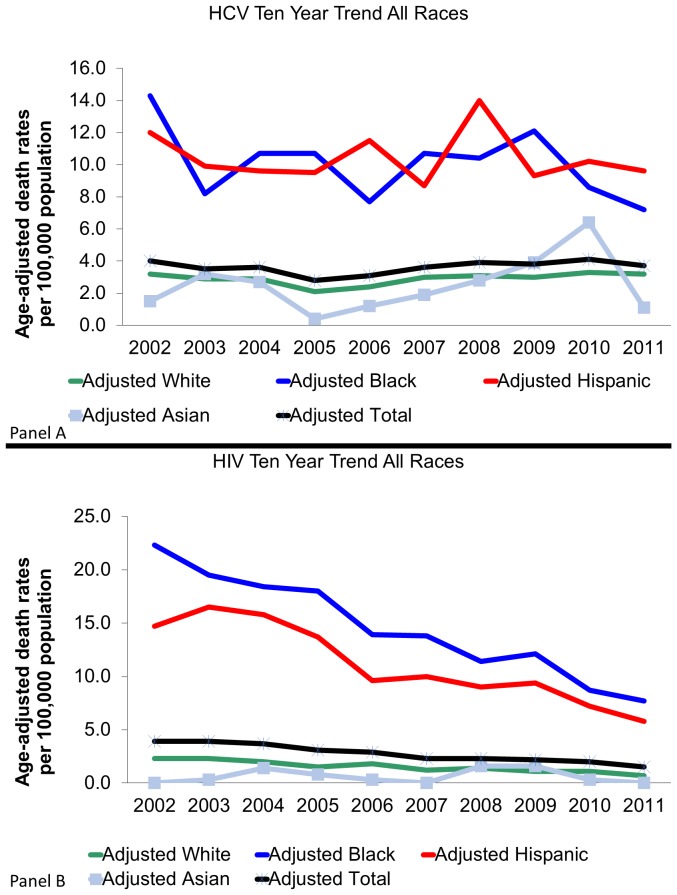
10 Year Age Standardized HCV and HIV/AIDS Mortality Trends per 100,000 by Race in MA. Panel A is Hepatitis C. Panel B is HIV/AIDS.

Fig. 5, Panels A and B illustrate age adjusted standardized mortality rates for HCV and HIV/AIDS respectively for each year in the ten-year period stratified by race groups as well as a total adjusted mortality trendline. As can be seen in Fig. 5A, the HCV mortality rates across all races remained relatively stable yet there is still a large disparity between White and Asian populations and Hispanic and Black populations. The trend in Fig. 5B for HIV/AIDS mortality differs slightly as the mortality rates remained relatively constant for White and Asian populations yet had a decline in Black and Hispanic populations.

### Spatial Cluster Analysis

**Figure 6:**
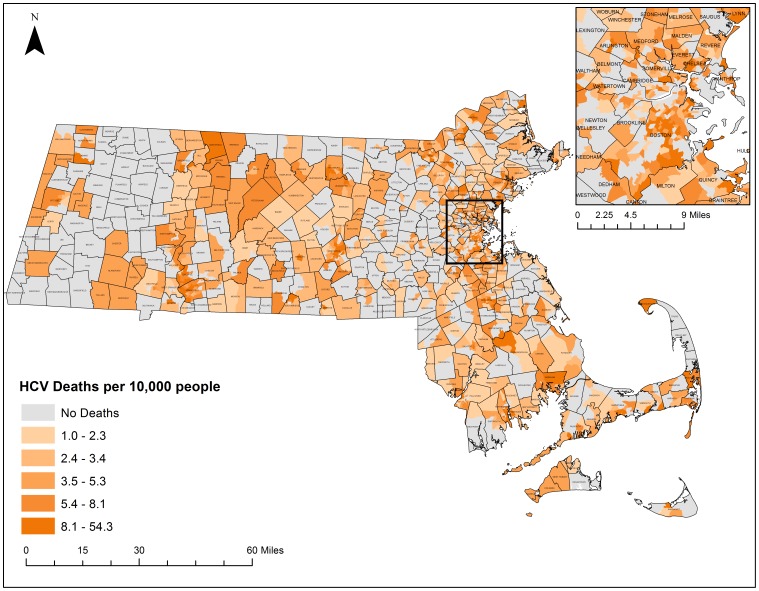
HCV Mortality rates by census tract, 2002–2011. Crude Mortality Rates were calculated based on the 2010 census population estimates at the census tract level for all-causes of HCV. Rates were classified by quintile. Shapefiles were provided by MassGIS, death data were provided by the Massachusetts Department of Public Health, and population estimates were provided by the US Census Bureau. NAD 1983 Massachusetts State Plain was used for projection. Maps created in ArcGIS 10.2.

**Figure 7:**
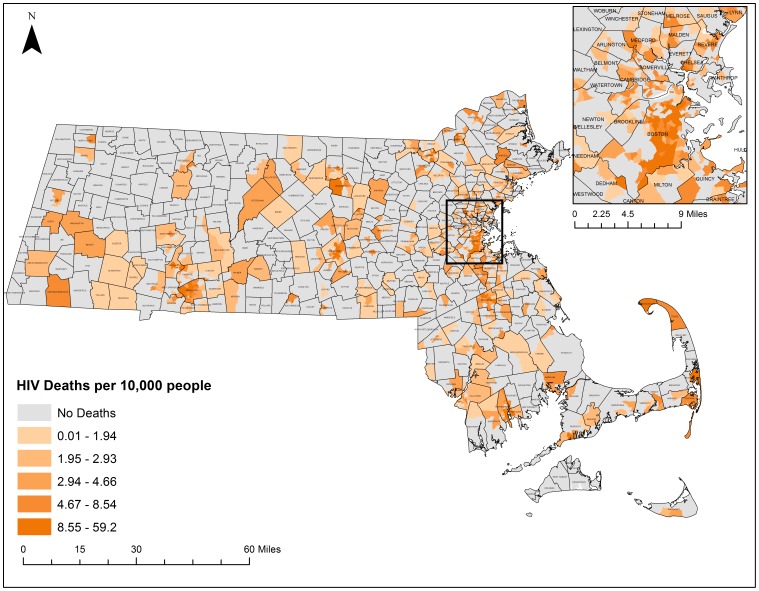
HIV/AIDS Mortality rates by census tract, 2002–2011. Crude Mortality Rates were calculated based on the 2010 census population estimates at the census tract level for all-causes of HIV/AIDS. Rates were classified by quintile. Shapefiles were provided by MassGIS, death data were provided by the Massachusetts Department of Public Health, and population estimates were provided by the US Census Bureau. NAD 1983 Massachusetts State Plain was used for projection. Maps created in ArcGIS 10.2.

Thematic maps displaying the geographic distribution of HCV and HIV/AIDS mortality can be found in Figs. 6 and 7 respectively. These maps present a descriptive look at the distribution of mortality at the census tract level with darker colors exhibiting higher mortality rates per 10,000 population. Both maps portray what appear to be clustering patterns of mortality. While these descriptive maps bring attention to several regions, statistically based spatial analyses are needed to determine whether significant clustering is occurring.

**Figure 8:**
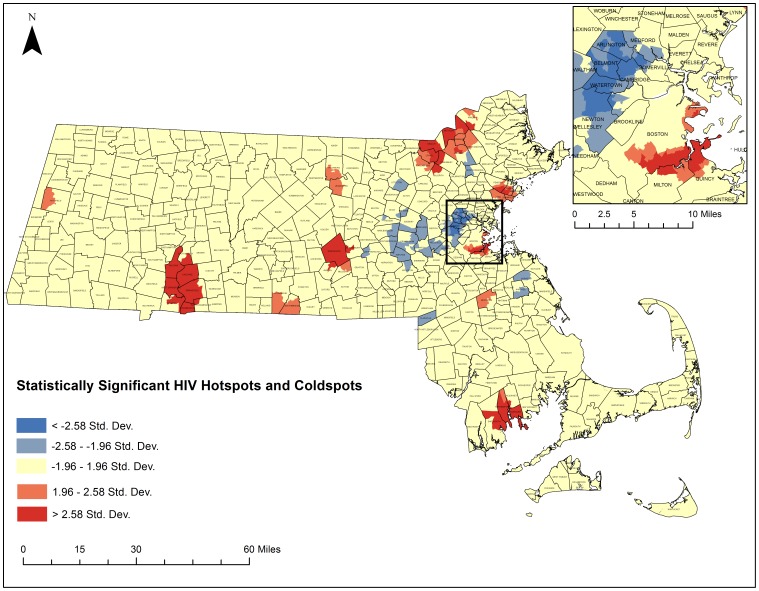
Statistically significant hotspots and coldspot clusters of HCV in Massachusetts census tracts between 2002–2011. A Getis Ord GI* test was run as specified in the five-step geoprocessing approach. Red areas represent statistically significant hotspots of all-cause HCV mortality. Blue areas represent statistically significant coldspots. Darker colors represent higher levels of significance. Shapefiles were provided by MassGIS, death data were provided by the Massachusetts Department of Public Health, and population estimates were provided by the US Census Bureau. NAD 1983 Massachusetts State Plain was used for projection. Maps created in ArcGIS 10.2.

**Figure 9:**
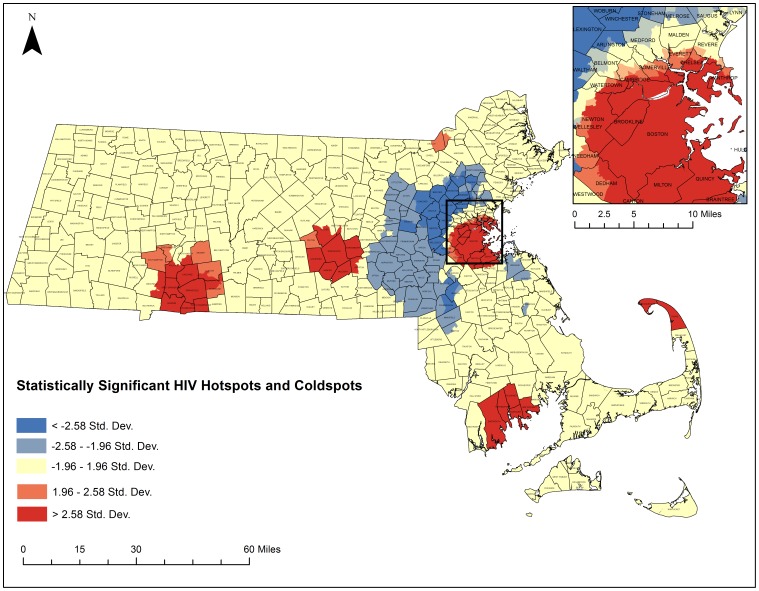
Statistically significant hotspots and coldspot clusters of HIV/AIDS in Massachusetts census tracts between 2002–2011. A Getis Ord GI* test was run as specified in the five-step geoprocessing approach. Red areas represent statistically significant hotspots of all-cause HIV/AIDS mortality. Blue areas represent statistically significant coldspots. Darker colors represent higher levels of significance. Shapefiles were provided by MassGIS, death data were provided by the Massachusetts Department of Public Health, and population estimates were provided by the US Census Bureau. NAD 1983 Massachusetts State Plain was used for projection. Maps created in ArcGIS 10.2.

The five-step geoprocessing approach was carried out for each disease of interest to identify hotspot clusters. From the original 1,471 census tracts, 1,373 were taken after removing those 1.5 standard deviations larger than the mean area and any remaining island census tracts. The average distance between census tracts was calculated to be 2,244 meters and the maximum distance was calculated to be 9,361 meters. After running incremental spatial autocorrelations for HIV/AIDS and HCV, HIV's ideal spatial scale was identified to be 12,000 meters while HCVs was identified to be 7,200 meters. These numbers were used to generate spatial weights matrices that were used to weight the final Getis-Ord Gi* analysis. Fig. 8 depicts the final hotspot analysis results for HCV and Fig. 9 highlights the final hotspot analysis results for HIV/AIDS.

Three hundred and thirty census tracts where located within statistically significant HCV hotspots and 110 census tracts were located within statistically significant HCV cold spot clusters. Five hundred census tracts were identified within statistically significant HIV/AIDS hotspots and 190 census tracts were located within statistically significant HIV/AIDS cold spots. Both diseases had clear regions throughout the state that exhibited hotspots and coldspots with common hotspots in South Boston, around New Bedford, Worcester, Springfield, and around the Merrimack Valley. Both had common cold spots in the Metrowest region. There were some differences in hotspot locations for each disease. HCV had several hotspots in more rural areas of the state that HIV/AIDS did not have. HIV/AIDS had significant clustering on Cape Cod, which was not present for HCV.

### Spatial Temporal Cluster Analysis

**Figure 10:**
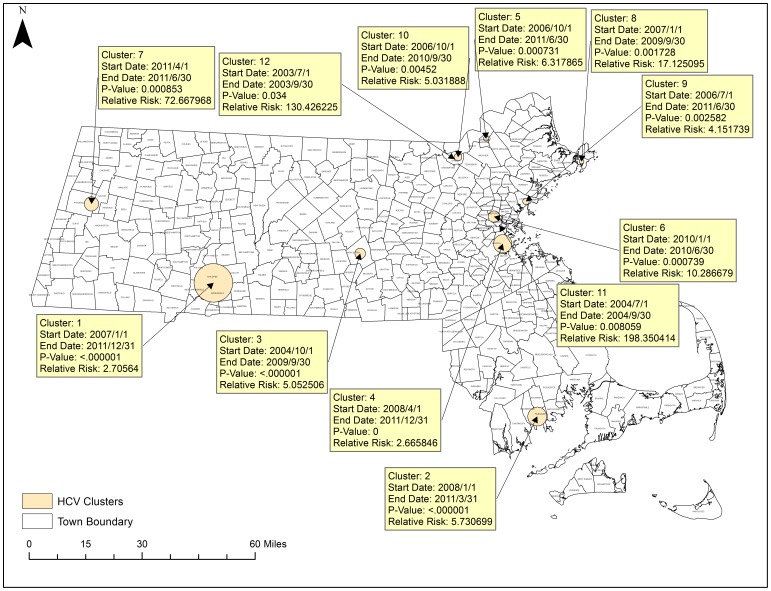
Statistically Significant Space-time and Spatial Trend Clusters in HCV. SatScan was used to test for spatial variation in temporal trends and for significant space-time clusters. Yellow boxes detail significant space-time clusters. Green boxes detail significant spatial variations in temporal trending. Shapefiles were provided by MassGIS, death data were provided by the Massachusetts Department of Public Health, and population estimates were provided by the US Census Bureau. NAD 1983 Massachusetts State Plain was used for projection. Maps created in ArcGIS 10.2.

Fig. 10 portrays a map of the results of the space-time cluster analysis and the trends in spatio-temporal trends analysis. There were 11 significant space-time clusters and 2 significantly different temporal trend clusters. Within the space-time clusters, the relative risk of mortality due to HCV is given compared to locations outside of the cluster during that same time period. For example, the risk of mortality due to HCV in Cluster 1 is 2.7 times that of the state mean in that geographic area of Springfield from January 2007 to December 2011. For the spatial variation in temporal trend clusters, the percent change in mortality rates within that cluster is provided compared to the expected external trend during that time period. For example, in Trend 1, in southeastern Massachusetts (Hyannis, Cape Cod) HCV mortality increased by 403% from 2002 to 2011 while the rest of the state increased on average at a rate of 4.8% during that time period. Of note, many of these clusters aligned with the spatial hotspots conducted previously such as the cluster in Springfield and the Cluster in Worcester. This cluster in Hyannis did not appear using spatial data alone and was only present when incorporating the time element.

Clusters are ordered from 1 to 12 by their statistical significance with Cluster 1 being the most significant. Some clusters have longer time periods, which likely represent longer-term trends in mortality. The most significant clusters occurred in Springfield where HCV mortality risk was 2.7 times that of the state average, and in New Bedford where HCV mortality risk was 5.73 times that of the rest of the state. Clusters with very large relative risks such as clusters 11 and 12 may be a result of a relatively small population within that geographic area which would inflate risk estimates.

**Figure 11:**
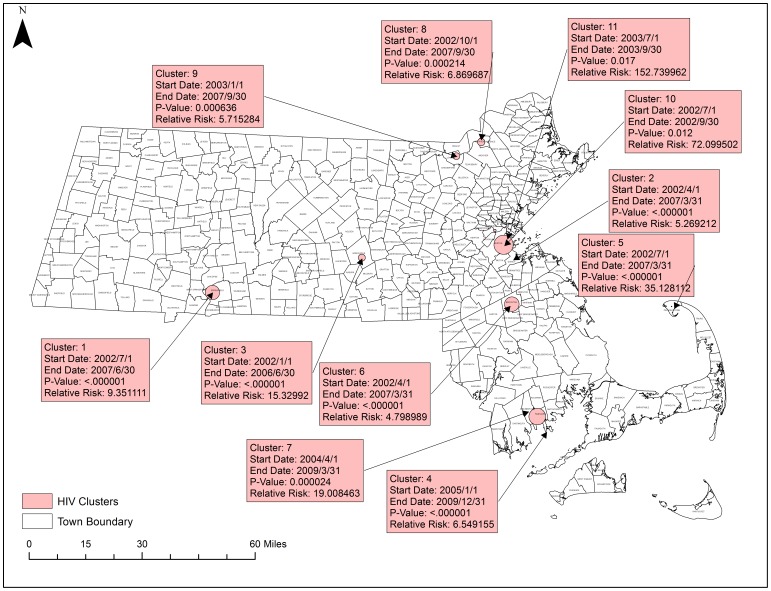
Statistically Significant Space-time clusters in HIV/AIDS. SatScan was used to test for spatial variation in temporal trends and for significant space-time clusters. Red boxes detail significant space-time clusters. There were no significant spatial variations in temporal trending. Shapefiles were provided by MassGIS, death data were provided by the Massachusetts Department of Public Health, and population estimates were provided by the US Census Bureau. NAD 1983 Massachusetts State Plain was used for projection. Maps created in ArcGIS 10.2.

Fig. 11 displays the 9 statistically significant space-time clusters for HIV/AIDS. There were no statistically significant spatial variation clusters in temporal trends. The most significant clusters were in Springfield with a relative risk of 9.35 and south of Boston with a relative risk of 5.26.

## Discussion

Our analysis finds several significant trends in HIV/AIDS and HCV mortality across the state of Massachusetts from 2002 to 2011. Overall, while total HIV/AIDS deaths are on a decline across the state, HCV mortality appears to be relatively stable. The observed stability of the HCV mortality rates over the ten year period differs from trends found in similar analyses which have found increasing mortality rates [4]. While the definitions of mortality were similar between these analyses, our study did not find a significant increasing trend in HCV mortality over the ten-year period. This being said, in Fig. 5, the overall trend did appear to increase. The difference in significance may be contributed to the use of Massachusetts death data alone which may be differential to the entire country taken in aggregate as Massachusetts has in the past experienced health outcomes greater than national averages [1].

Trends vary among ethnicity/race groups. For HIV/AIDS, it appears that much of the decline in annual mortality across the state can be attributed to steep declines in HIV/AIDS among Hispanic and black populations with relatively constant rates among White and Asian populations.

Multiple cause mortality data helps to present a more complete picture of HIV/AIDS and HCV mortality trends and clustering. Within mortality data, HCV more typically appears as a contributing cause of death rather than an underlying cause of death. As a result, traditional underlying cause reporting of death may severely underestimate the burden HCV mortality has on the population. HIV/AIDS conversely appears to be primarily reported as an underlying cause of death. This may be the result of either HIV/AIDS taking higher reporting priority by whomever is filling out the death certificate, or that SuperMICAR, the program that helps select underlying and contributing cause of death, may more frequently assign HIV/AIDS as an underlying cause of death, rather than a contributing cause, due to the algorithms it uses in selection. In either case, the full burden of HIV/AIDS may be adequately captured through underlying cause methodologies alone. As shown in Fig. 2, HCV deaths may also be severely underreported.

Racial and ethnic disparities exist in HIV/AIDS and HCV mortality in Massachusetts. The adjusted mortality rates for HIV/AIDS and HCV among black and Hispanic populations are up to 5 times as high as the rates in white and Asian populations. These disparities should be taken into consideration when designing programs for the long-term control of those two conditions.

For HIV/AIDS and HCV, most deaths occurred among those ages 45–64. It is currently believed that ongoing transmission of HIV/AIDS and HCV is primarily taking place at higher rates among adolescents and young adults across the state [20] which seems to differ from the current trend in mortality. Given the age distribution, the multiple cause mortality featured in this study are likely among people who have been living with HIV and HCV as a chronic condition for longer periods of time. It is important to note that where the clusters currently are taking place may not be clusters of where infection took place. Incidence data may provide greater insight into current transmission patterns than mortality data. Despite the limitations in measuring mortality over a potentially large latency period, analyses focused on mortality data are still of public health importance as the populations identified by this study can be targeted for the allocation of resources for long term supportive care rather than programs designed for the prevention of further transmission.

There exist clear regions of spatial clustering of disease. Of particular note are the South Boston, the Merrimack Valley, Springfield, Worcester, and New Bedford Regions of the state. The clustering of high mortality rates are too significant for chance alone to explain their existence and further research should be directed at these regions to examine what underlying factors have helped contribute to these patterns. Poverty levels and racial distributions may play a role however the hotspot cluster analysis method cannot build these additional variables into the model so more research and statistical modeling is needed. Spatial regression analyses and multivariate statistical models could improve our understanding of the individual, community, and structural factors associated with HIV/AIDS and HCV mortality hotspots. Nonetheless, the hotspot cluster maps present a robust look at where mortality rates are the highest or lowest which may help to inform resource allocation, and targeting of public health interventions.

Space-time clustering did occur significantly across the state for HIV/AIDS and HCV mortality to a degree that cannot be explained by chance alone. At the time of submission, these trends have not been characterized in Massachusetts before, nor have many studies been conducted across the state using multiple cause methodologies. The clusters found in this analysis can help characterize the burden of mortality. With the awareness of where HIV/AIDS and HCV mortality is occurring, resources can be better diverted and the burden of mortality can be addressed. This study supports the belief that most HIV/AIDS and HCV mortality is occurring in more urban areas with cities presenting as the primary locations of clusters. However, key clusters for HCV existed in both the SatScan and Getis-Ord GI* analyses in several suburban and rural locations as well. More research is needed to determine whether similar suburban and rural clustering is evident in prevalence data and incidence data, which is more reflective of recent disease transmission. If such clustering patterns are evident in prevalence and incidence data, there may be unmet need for more HIV and HCV prevention, testing, treatment, and care programs in suburban and rural locations across the state.

More research should be focused on the time periods and locations of these space-time clusters as well as locations of temporal trend clusters to see what mechanisms may have played a role in these periods of increased mortality. The time element allows for us to identify more precisely what policy, demographic, structural, and behavioral shifts may have contributed to these changes in mortality patterns, and can help public health officials to better target interventions to address these infections. The systematic and rigorous approaches used to identify clusters in this study help to provide a clearer picture of HIV and HCV patterns in the state which merit further attention in order to more effectively prevent future HIV/AIDS and HCV related mortality.

### Limitations

There are several important limitations of note that must be taken into account when considering the results of this study. First, there are known misclassification in death certificates. Many clinicians are not well trained in the completion of death certificates and, as a result, underlying and contributing causes of death may not be properly coded. Additionally, in the absence of a family member, often the person filling out a death certificate will assume the racial identity of the decedent, which can be problematic in particular for smaller racial groups such as American Indian, Asians and Hispanics, which may be underrepresented. Death data offers a very limited view at race and is unable to offer insight into potential disparities between more specific racial and ethnic groups.

When using SatScan, there is currently no defined method to select a proper scanning window and timeframe. As a result, an assumption must be made as to the proper size of a potential outbreak. We selected a scanning window of 5% to ensure the most granular clusters, however there are limited methods through which to test the validity of such an assumption. Furthermore, despite the use of p-value cutoffs, this analysis does not preclude the effect that chance alone may have influenced some clusters.

This analysis using Hotspot Cluster Analysis and SatScan does not take into account all possible confounders. The clustering that has taken place may be largely driven by factors such as socio-economic status, low income housing affordability, and location of treatment and support centers among others. Despite this limitation, the usefulness of this analysis Is not greatly diminished as if taken along with future research into the effects of socio-economic status and other factors, there may be significant clustering that does not overlap giving these results utility.

In addition, when using multiple cause data there is a potential for a contributing cause to bear no actual impact on decedent's death. For example, if a decedent who had HIV was killed in a car accident, he/she may have been included in the mortality data as an HIV/AIDS death while the HIV infection actually did not contribute to the actual death. In such a scenario, the death certificate would still provide valuable information on the demographics of those living with HIV/AIDS, but may skew mortality results. However, we do not anticipate that such misclassification would be differential with regard to our outcome of interest.

## Conclusions

This study represents a novel approach to the investigation of trends in infectious disease mortality in Massachusetts. To our knowledge, multiple cause methodologies have not been employed previously for investigating ten years' worth of infectious disease data in the state. Additionally, to our knowledge, this is the first application of multiple cause methodologies to both the 5-step geoprocessing hotspot analysis approach and space-time cluster analysis in SatScan. The results have yielded insight on significant patterns and trends viewed demographically, temporally, and spatially in infectious disease mortality across the state. More attention and resources for HIV/AIDS and HCV prevention, treatment, and care should be paid to minority groups and groups with elevated behavioral risks (e.g., injection drug users, men who have sex with men, high-risk heterosexuals) in the regions of significant disease clustering. Multiple cause analysis should be conducted more extensively across the state in order to assess further patterns that might exist in a wide variety of diseases. As the data for HCV indicate, looking at underlying cause data alone is not sufficient to evaluate the full extent of related mortality throughout the state.
